# Puzzling painful nodules on back: a case report

**DOI:** 10.11604/pamj.2022.42.203.34093

**Published:** 2022-07-14

**Authors:** Meghana Pendam, Bhushan Madke, Samyak Ganjre, Meenakshi Chandak, Adarsh Lata Singh

**Affiliations:** 1 Department of Dermatology, Venereology and Leprosy, Jawaharlal Nehru Medical College, Datta Meghe Institute of Medical Science, Wardha, Maharashtra, India

**Keywords:** Piloleiomyoma, arrector pili muscle, calcium channel blocker, painful skin tumour, case report

## Abstract

Cutaneous leiomyomas are known to arise from three locations viz, arrector pili muscle, dartos muscle in the scrotal sac, and smooth muscle layer of blood vessels. Cutaneous piloleiomyoma (arising from arrector pili) is an uncommon benign skin tumor presenting clinically as a single or numerous painful nodules. They can either appear diffuse or zosteriform/segmental in rare cases. We report an unusual case of multiple tender papulonodular lesions on the right infrascapular region. Histology from one of the nodules showed a dermal tumor consisting of interlacing fascicles of spindle cells, consistent with the diagnosis of piloleiomyoma. We prescribed our patient with oral calcium channel blocker, amlodipine, to provide symptomatic relief from pain and which can be considered used it to treat similar cases.

## Introduction

Smooth muscle neoplasms called leiomyomas are benign soft tissue neoplasms. They have the tendency to grow where there is smooth muscle [1,2]. They appear as dermal papules or nodules that are pink or skin-colored and adhere to the skin. Piloleiomyomas are frequently found in clusters, with each lesion varying in size from a few millimeters to a few centimeters. The distribution is most commonly clustered, linear, or along lines of Blaschko's [3]. Congenital piloleiomyomas have also been reported, but are extremely rare [4]. It can also be hypothesized that genetic mosaicism is a clinical manifestation of a genetically programmed clone of changed cells [5]. Here, we report a case of a 31-year-old male with painful nodules on the back who was treated with an oral calcium channel blocker.

## Patient and observation

**Figure 1:**
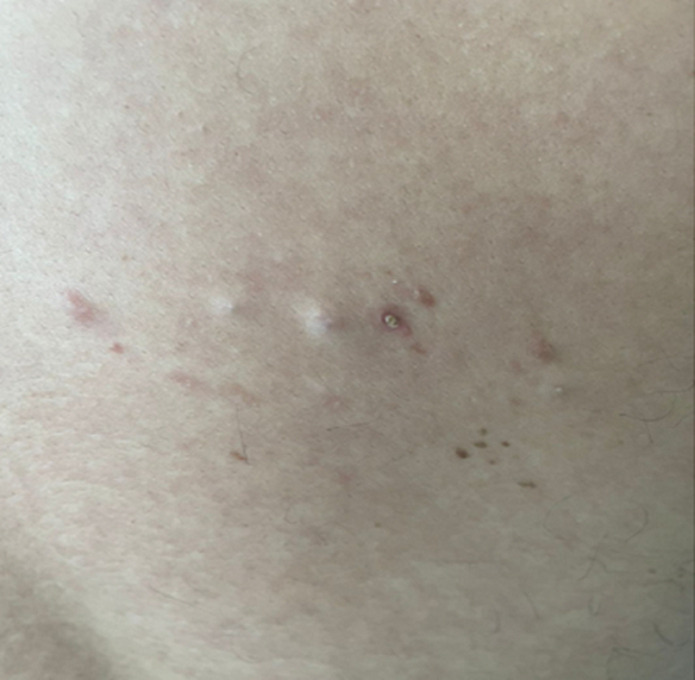
nodules on the right infrascapular region

**Patient information:** a 31-year-old male came with a complaint of a few raised skin-colored painful lesions on the right side of the back for six months. The patient noticed the appearance of a single nodule on the right infrascapular area. Over of period of six months, he noticed similar skin nodules which were painful and tender to touch (Figure 1).

**Clinical findings:** cutaneous examination showed skin colored to bluish hue nodules of size ranging from 3 cm to 4 cm (approximately) present on right infrascapular region.

**Timeline of the current episode:** they were complaints of mild pain 6 months back which aggravated for 2 weeks.

**Figure 2:**
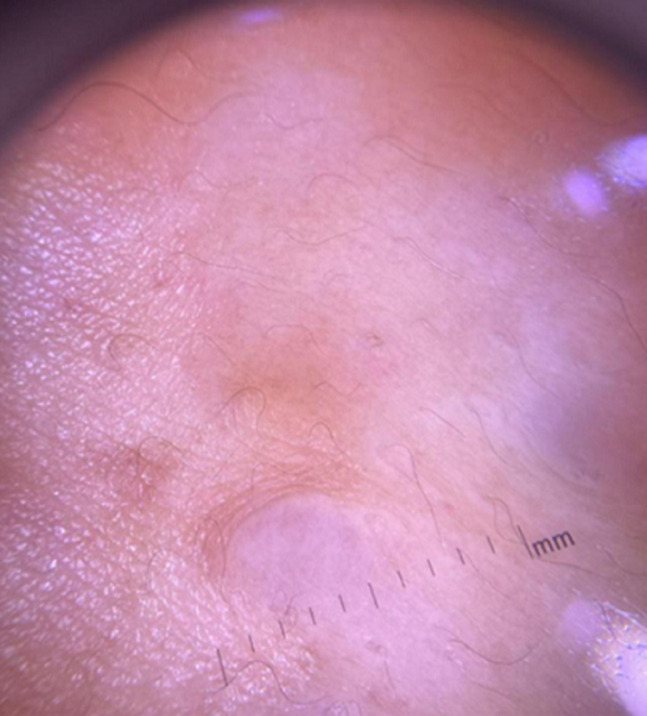
contact dermoscopy of the nodules showing central hypopigmented area with peripheral delicate reticular network

**Figure 3:**
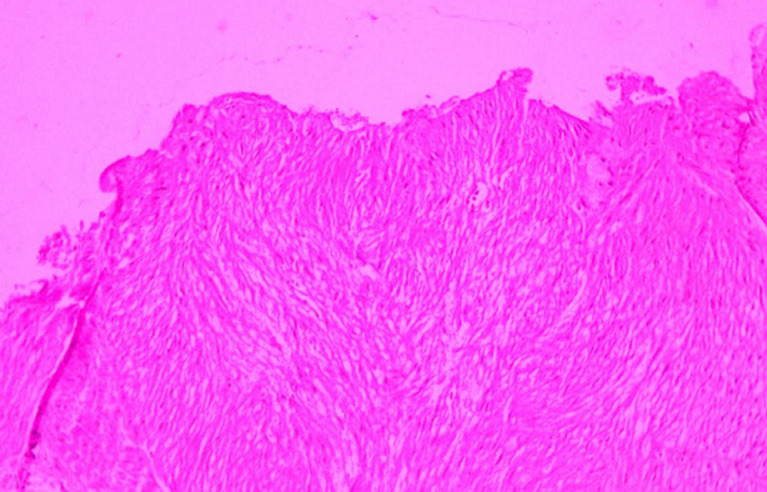
H&E-stained section showing interlacing bundles of spindle cells with moderate amount of eosinophilic cytoplasm and perinuclear halos

**Diagnostic assessment:** contact dermoscopy of the nodules showed the central hypopigmented area with peripheral delicate reticular network (Figure 2). Complete hemogram, blood sugar level, and serum biochemistry were within the normal reference range. After informed consent, a 4 mm punch biopsy was done from one of the representative nodules. Hematoxylin and eosin (H&E)-stained section showed interlacing bundles of spindle cells with moderate amount of eosinophilic cytoplasm and perinuclear halos (Figure 3) suggestive of piloleiomyoma.

**Therapeutic interventions:** on the basis of clinic-pathological correlation, we made a diagnosis of segmental piloleiomyoma. We could not perform mutation analysis for the fumarate hydratase gene. The patient was prescribed oral amlodipine 10 mg once a day for symptomatic relief of pain and tenderness.

**Follow-up and outcome of interventions:** the H&E-stained section showed interlacing bundles of spindle cells with a moderate amount of eosinophilic cytoplasm and perinuclear halos suggesting piloleiomyoma for which the patient was advised Tab. Amlodipine 10 mg once a day, which has shown significant relief from pain.

## Discussion

Cutaneous leiomyomas account for only about 5% of leiomyomas three forms of cutaneous leiomyomas viz. Piloleiomyoma, angioleiomyoma, and genital leiomyoma arise from arrector pili muscle, vascular smooth muscle and, dartos muscle respectively piloleiomyoma are one of the differentials for painful skin tumors and commonly occurs in the age range of 10 to 30 years. On the extensor surface of the limbs, trunk, face, and neck, they appear as skin-colored to erythematous papulonodular lesions that can be single or many. Piloleiomyomacan be distributed in a grouped, diffuse or segmental pattern [6]. Multiple cutaneous and uterine leiomyomatosis (MCUL) is an autosomal-dominant trait in which multiple cutaneous and uterine leiomyomata are observed to be connected with uterine leiomyomata [7]. Two theories have recently been proposed to explain the occurrence of segmental leiomyomas. Heterozygosity of a postzygotic mutation causes segmental skin lesions in type-1 segmental leiomyoma, which are comparable to non-mosaic phenotype. A postzygotic mutation in a heterozygous embryo causes type-2 segmental leiomyoma, which is characterized by a conspicuous pattern of segmental lesions layered on the underlying disease's normal phenotype [8]. Reed´s syndrome also known as familial leiomyomatosis cutis et uteri, is characterized by the presence of both cutaneous and uterine leiomyomas. Females who are afflicted are more prone than the general population to have uterine leiomyomas that are larger, more numerous, and emerge earlier. Reed´s syndrome occurs due to mutation of the gene located on 1q42.3-43, which in the Krebs cycle, the fumarate hydrates enzyme catalyzes the conversion of fumarate to malate. Excision is the therapy of choice for single cutaneous leiomyoma, however, in widespread involvement primary excision and grafting may carry a risk of morbidity and scarring. Calcium channel blockers, adrenoceptor blocking agents, nitrates, analgesics, antidepressants, and gabapentin are some drugs used in medical management. Since piloleiomyoma consists of smooth muscle tissue, calcium channel blockers have been tried with variable success. Nifedipine, a calcium channel blocker, has been previously used to treat painful cutaneous leiomyomas with good outcomes. Amlodipine was prescribed to our patient since it has a lower risk of adverse effects (headache and flushing) than nifedipine. Cryosurgery, carbon dioxide laser, and botulinum toxin are some additional therapy modalities that have been tried [9,10].

## Conclusion

Leiomyomas are rare benign tumors that derive from the proliferation of smooth muscle cells. They present as firm, skin-colored, light brown, or reddish brown papules or nodules for which skin biopsy can be done for confirmation of diagnosis and can be treated using Tab. Amlodipine 10 mg with regular monitoring of blood pressure, which provides significant relief from pain.
